# Effects of Different Lengths of a Nucleic Acid Binding Region and Bound Nucleic Acids on the Phase Behavior and Purification Process of HBcAg Virus-Like Particles

**DOI:** 10.3389/fbioe.2022.929243

**Published:** 2022-07-01

**Authors:** Angela Valentic, Jakob Müller, Jürgen Hubbuch

**Affiliations:** Institute of Process Engineering in Life Sciences—Section IV: Biomolecular Separation Engineering, Karlsruhe Institute of Technology (KIT), Karlsruhe, Germany

**Keywords:** nanocarrier (nanoparticle), gene therapy, downstream processing, virus-like particles, nucleic acid binding, disassembly, phase behavior, capsid stability

## Abstract

Virus-like particles (VLPs) are macromolecular structures with great potential as vehicles for the targeted administration of functional molecules. Loaded with nucleic acids, VLPs are a promising approach for nanocarriers needed for gene therapy. There is broad knowledge of the manufacturing of the truncated wild-type lacking a nucleic acid binding region, which is mainly being investigated for vaccine applications. Whereas for their potential application as a nanocarrier for gene therapy, hepatitis B core antigen (HBcAg) VLPs with a nucleic acid binding region for efficient cargo-loading are being investigated. VLP structure, loading, and phase behavior are of central importance to their therapeutic efficacy and thereby considerably affecting the production process. Therefore, HBcAg VLPs with different lengths of the nucleic acid binding region were produced in *E. coli*. VLP attributes such as size, zeta potential, and loading with host cell-derived nucleic acids were evaluated. Capsid’s size and zeta potential of the VLP constructs did not differ remarkably, whereas the analysis of the loading with host cell-derived nucleic acids revealed strong differences in the binding of host cell-derived nucleic acids dependent on the length of the binding region of the constructs, with a non-linear correlation but a two-zone behavior. Moreover, the phase behavior and purification process of the HBcAg VLPs as a function of the liquid phase conditions and the presence of host cell-derived nucleic acids were investigated. Selective VLP precipitation using ammonium sulfate was scarcely affected by the encapsulated nucleic acids. However, the disassembly reaction, which is crucial for structure homogeneity, separation of encapsulated impurities, and effective loading of the VLPs with therapeutic nucleic acids, was affected both by the studied liquid phase conditions, varying pH and concentration of reducing agents, and the different VLP constructs and amount of bound nucleic acids, respectively. Thereby, capsid-stabilizing effects of the bound nucleic acids and capsid-destabilizing effects of the nucleic acid binding region were observed, following the two-zone behavior of the construct’s loading, and a resulting correlation between the capsid stability and disassembly yields could be derived.

## Introduction

Nucleic acid-based therapeutics, such as mRNA, antisense oligonucleotides, or small interfering RNA, are investigated for selective and efficient therapies used for a broad spectrum of medical fields such as immunotherapy, oncology, and infectious diseases (Burnett et al., 2011; Chen and Zhaori, 2011; Sahin et al., 2014; Sharma et al., 2014; Nikam and Gore, 2018; Zhou et al., 2019). Lately, nucleotide-based vaccines have proven their efficacy as vaccines against infectious diseases and their vast potential for fast development up to large-scale manufacturing (Zhong et al., 2018; Fortner and Schumacher, 2021): First, vaccines against the coronavirus disease 2019 authorized for emergency use by the FDA and EMA were two mRNA-based vaccines (Fortner and Schumacher, 2021). Lipid nanoparticles were used for packaging and delivery (Hou et al., 2021). Lipid-based nanocarriers have been studied intensively in recent years (Blasi et al., 2007; Xue et al., 2015; Kulkarni et al., 2018; Pardi et al., 2018; Aldosari et al., 2021). However, there are still challenges such as efficient tissue targeting and lipid toxicity (Aldosari et al., 2021). A promising alternative is virus-like particles (VLPs) for the targeted delivery of small molecules, proteins, and nucleic acids (Rohovie et al., 2017). The main advantages over lipid-based nanocarriers are the possibility of surface modification on a genetic level or post-translational modification for effective tissue targeting and precise and uniform structures with large loading capacities, depending on the type of VLP (Rohovie et al., 2017). Several VLPs studies showed the ability to pack RNA (Porterfield et al., 2010; Li et al., 2013; Petrovskis et al., 2021), protect (Fang et al., 2018), and deliver nucleic acids (Cooper and Shaul, 2005; Choi et al., 2013).

**FIGURE 1:**
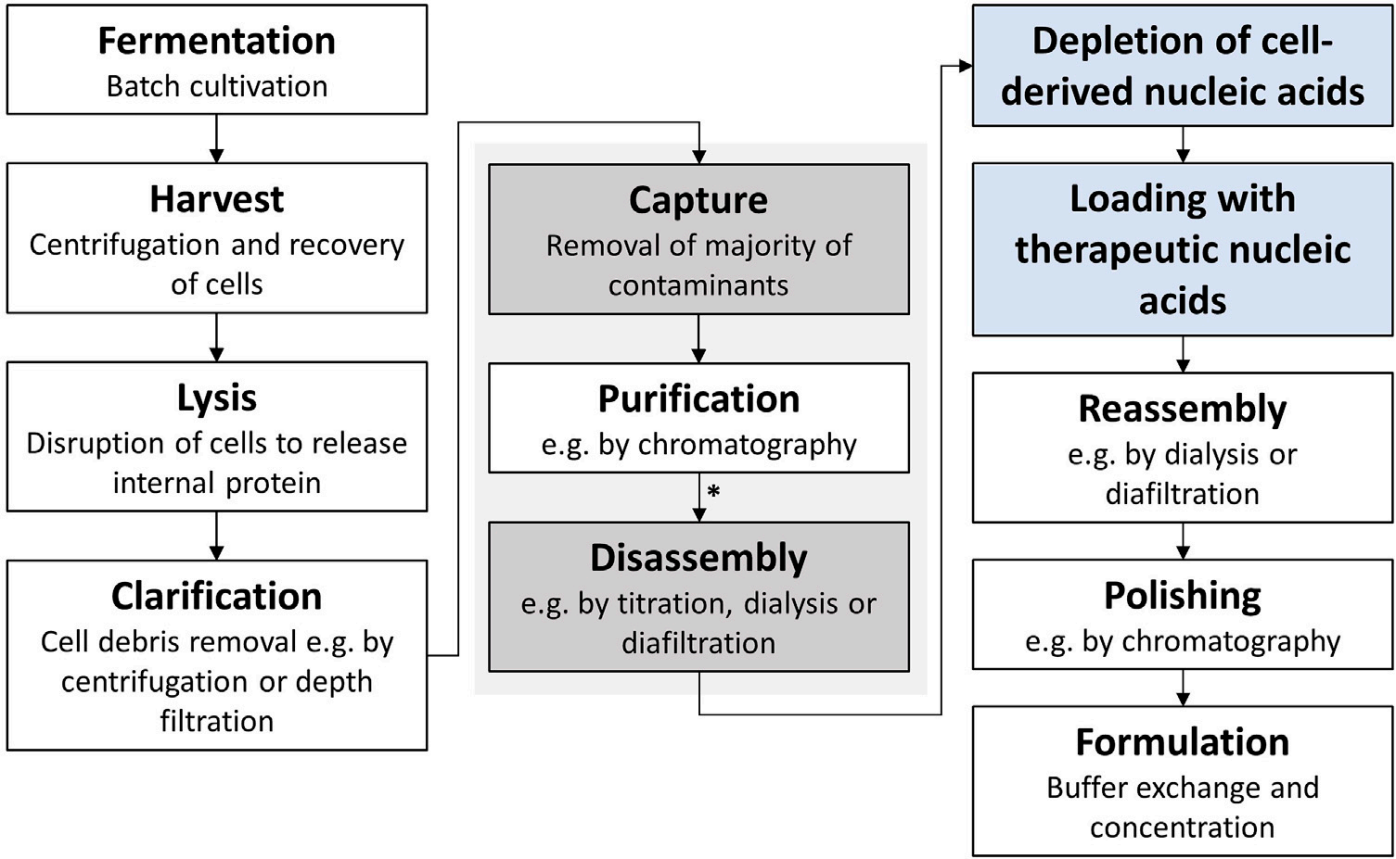
Typical HBcAg VLP purification process (Hillebrandt et al., 2021) with additional process steps for application as a transport vector for nucleic acids highlighted in blue. The central purification steps captured by selective precipitation and disassembly, investigated in this study, are highlighted in grey. HBcAg VLP construct characterization was conducted after purification by CaptoCore 400 chromatography and dialysis (marked with an asterisk *). HBcAg, hepatitis B core antigen; VLP, virus-like particle.

However, effective VLP downstream processing, especially including loading with the therapeutic nucleic acids, is still a challenge. Following the intracellular formation of VLPs in an expression system, such as *E. coli*, yeast, or plant cells (Effio and Hubbuch, 2015), a commonly used VLP purification process consists of cell lysis, ultracentrifugation or precipitation, disassembly and reassembly, and polishing and formulation (McCarthy et al., 1998; Zhao et al., 2012; Hillebrandt et al., 2020; Zhang et al., 2021). Traditional processes utilize size-exclusion chromatography (SEC) and ultracentrifugation (Yoon et al., 2013). However, there have been recent advances in the processing using ultrafiltration-based unit operations, exploiting the size differences between impurities, VLP subunits, and VLP capsids, investigating VLPs for vaccine applications (Negrete et al., 2014; Carvalho et al., 2019; Rüdt et al., 2019; Hillebrandt et al., 2020; Hillebrandt et al., 2021). However, VLPs, being developed for nucleic acid delivery, have an even more complex purification process due to the need for an additional loading step with therapeutic nucleic acids. A typical production process is shown in Figure 1. To be able to make use of these recent developments toward effective and large-scale purification processes for VLPs, deeper knowledge of VLP characteristics and influencing factors, such as encapsulated nucleic acids, on the purification process needs to be derived.

Among the candidates investigated for their use as nanocarriers for gene therapy is hepatitis B core antigen (HBcAg) VLP. Typically, HBcAg VLPs are mixed with therapeutic nucleic acids in a disassembled state and are then reassembled with the nucleic acids encapsulated in the VLP capsids (Porterfield et al., 2010; Petrovskis et al., 2021; Zhang et al., 2021). For an effective loading of the VLPs, often the naturally occurring full-length nucleic acid binding region of the wild-type HBcAg protein (Nassal, 1992; Zhang et al., 2021), further referred to as Cp183 (amino acids 1–183), and variants with few replaced amino acids (Porterfield et al., 2010; Strods et al., 2015) are employed. Moreover, HBcAg VLP constructs with different lengths of this naturally occurring arginine-rich nucleic acid binding region are investigated (Newman et al., 2009; Liu et al., 2010; Sominskaya et al., 2013; Petrovskis et al., 2021). One major challenge for the purification process arising with the nucleic acid binding region is the undesired association and encapsulation of host cell-derived nucleic acids during spontaneous assembly throughout the expression of HBcAg VLP protein subunits, further referred to as dimers. Various techniques such as lithium chloride precipitation (Porterfield et al., 2010), alkaline treatment (Strods et al., 2015), and enzymatic treatment (Newman et al., 2009), sometimes coupled with affinity chromatography (Zhang et al., 2021), are applied during the purification process to separate the *E. coli* derived nucleic acids. Furthermore, the encapsulated nucleic acids appear to have an influence on the purification process and especially on the disassembly reaction. However, the disassembly reaction is not only crucial for separating bound nucleic acids (Porterfield et al., 2010; Strods et al., 2015; Zhang et al., 2021) and enabling effective loading with therapeutic nucleic acids but also for separating encapsulated impurities (Hillebrandt et al., 2021) and improving structural integrity (Zhao et al., 2012).

Purification processes for the wild-type HBcAg VLP construct with the full-length nucleic acid binding region (Cp183) and the arginine-rich C-terminal region truncated HBcAg VLP construct (Cp149) with amino acids 1 to 149 (Zlotnick et al., 1996) were described earlier (Porterfield et al., 2010; Zhang et al., 2021). In addition to the varying behaviors of the different constructs during purification, solubility issues for the Cp183 were reported (Porterfield et al., 2010). Moreover, next to Cp183 with the full-length nucleic acid binding region, it was shown that constructs with different lengths of the binding region can be successfully expressed in *E. coli*, purified and packed with therapeutic nucleic acids (Petrovskis et al., 2021), but with a set purification process and main focus on the selection of construct variants. However, a study on HBcAg VLPs without nucleic acid binding region demonstrated a distinct dependency of the disassembly reaction on the examined HBcAg VLP constructs (Hillebrandt et al., 2021). Thereby, for the wild-type truncated HBcAg protein (Cp149) and a construct with a chimeric epitope displayed on the surface of the VLP capsids, different rates, and optimal liquid phase conditions for the disassembly reaction were determined. Moreover, for a series of HBcAg VLP constructs with various lengths of the nucleic acid binding region, its influence on the VLP capsid stability was shown (Newman et al., 2009). This might have major effects on the phase behavior (in which a distinction is made between HBcAg dimers, capsids, or aggregated proteins) and the overall purification process.

This points toward a substantial influence of different lengths of the nucleic acid binding regions and encapsulated nucleic acids on the purification process of HBcAg VLPs. A nucleic acid binding region appears to be necessary to encapsulate a considerable amount of nucleic acids (Zhang et al., 2021), but examining different lengths might be useful to overcome challenges during the purification process such as the mentioned solubility issues for the construct Cp183 (Porterfield et al., 2010). Deeper knowledge of the influence of the nucleic acid binding region and bound nucleic acids on early downstream purification steps such as precipitation, re-dissolution, and especially the disassembly step needs to be derived to enable successful loading with therapeutic nucleic acids. This is relevant to prospectively enabling effective manufacturing of HBcAg VLPs for nucleic acid delivery.

In this study, we investigated six different HBcAg VLP constructs with different lengths of the nucleic acid binding region regarding their size, charge, and loading with host cell-derived nucleic acids derived from expression in *E. coli*. We further determined the effects of the various constructs and bound nucleic acids on the precipitation, re-dissolution, and disassembly steps of the purification process. A series of ammonium sulfate concentrations for precipitation and various buffer compositions for re-dissolution were assessed to show possible deviations between the different constructs. For the disassembly reaction, several liquid phase conditions with varying pH and urea concentrations were systematically examined to compare the disassembly behavior of the various constructs and evaluate the influence of the nucleic acid binding region and encapsulated nucleic acids on this phase behavior of the VLPs. Thereby, the results of this study demonstrated 1) the dependency of the phase behavior of the VLPs on the capsid stability, likewise affected by a complex interplay of the nucleic acids binding region and bound nucleic acids within the HBcAg proteins, and 2) its major influence on the disassembly reaction as one crucial purification step for VLP production.

## Materials and Methods

### Materials and Buffers

If not stated otherwise, all chemicals were purchased from Merck (Darmstadt, Germany). Solutions and buffers were prepared with ultrapure water (PURELAB Ultra, ELGA LabWater) and filtered through a 0.2 µm pore-size cellulose acetate filter (Pall Corporation, Port Washington, NY, United States). Buffers were pH-adjusted with 32% HCl. The lysis buffer consisted of 50 mM Tris, 100 mM NaCl, and 1 mM EDTA (AppliChem, GmbH, Darmstadt, Germany) at pH 8. As wash buffer, lysis buffer was adjusted to 1 M (NH_4_)_2_SO_4_ (AppliChem, Darmstadt, Germany) and 0.25% (v/v) polysorbate 20 (AppliChem, Darmstadt, Germany) with stock solutions of 4 M (NH_4_)_2_SO_4_ and 10% (v/v) polysorbate 20, respectively. The re-dissolution buffer was 50 mM Tris, 150 mM NaCl, and pH 7.2 for all experiments. 'For the disassembly screening, a 50 mM Tris, 10 M urea stock solution was used to dilute the protein solution in the re-dissolution buffer to the desired urea concentration.

### Cloning of Cp154, Cp157, Cp164, Cp167, and Cp183

Prof. Adam Zlotnick (Indiana University Bloomington, United States) provided the expression vector for the C-terminally truncated wild-type HBcAg with a sequence of 149 amino acids. Based on this Cp149 expression vector, plasmids coding for VLP constructs with different lengths of the nucleic acid binding region were produced. The amino acid sequences introduced as C-terminal nucleic acid binding regions are listed in Table 1. The plasmids coding for Cp154, Cp157, Cp164, and Cp167 were made by modifying the pET11c plasmid coding for Cp149, whereby the bp sequences encoding for the C-terminal amino acids were inserted. The regions encoding for Cp149 on the pET11-based vector were amplified using overlapping oligonucleotides to introduce site-directed mutagenesis using the polymerase chain reaction (PCR). The wild-type HBcAg Cp183 was obtained by amplifying the Cp167 plasmid in the same manner. The respective forward and reverse primers used for the PCR reaction for the production of the different constructs can be found in Supplementary Material S1. Amplification was performed with PCRBio HiFi polymerase (Nippon Genetics Europe GmbH, Düren, Germany). The PCR template and product were digested using DpnI (New England BioLabs, Ipswitch, MA, United States), purified by native gel electrophoresis, and extracted using a Wizard SV gel and PCR clean-up kit (Promega, Madison, WI, United States). Ligation was accomplished with the Gibson Assembly Master Mix (New England BioLabs, Ipswitch, MA, United States), and the new vectors were transformed into BL21 [DE3] cells (New England Biolabs, Ipswitch, MA, United States).

**TABLE 1:** Amino acid sequences in one-letter code of arginine-rich nucleic acid binding region in HBcAg VLP constructs. Residues are located C-terminally at the core domain with 149 amino acids (Crowther et al., 1994); HBcAg, hepatitis B core antigen; VLP, virus-like particle.

Construct	Amino acid sequence
Cp154	RRRGR
Cp157	RRRGRSPR
Cp164	RRRGRSPRRRTPSPR
Cp167	RRRGRSPRRRTPSPRRRR
Cp183	RRRGRSPRRRTPSPRRRRSQSPRRRRSQSRESQC

### Intracellular Formation and Purification of VLPs

VLPs were overexpressed in *E. coli* using a TB-based auto-induction medium and liberated by cell lysis directly as previously described (Hillebrandt et al., 2020). For selective VLP precipitation, the filtered lysate was diluted with (NH_4_)_2_SO_4_ and polysorbate 20 stock solutions, adjusted to 1 M (NH_4_)_2_SO_4_ and 0.25% (v/v) polysorbate 20, and stirred for 2 h at 4°C. The solution was spun down at 17,000 rcf for 30 min in a centrifuge 5810 R (Eppendorf, Hamburg, Germany). The supernatant was discarded while the pellet was resuspended with wash buffer and incubated at 10 rpm at room temperature in an overhead shaker LD-79 (Labinco, Breda, Netherlands) for 10 min, centrifuged with the identical settings, and the supernatant was discarded. The pellet was resuspended with re-dissolution buffer and stirred at 150 rpm overnight at 4°C. The solution was centrifuged at 1700 rcf for 30 min and filtered with 0.2 µm cellulose acetate syringe filters. After a dialysis step with 3.5 kDa molecular weight cut-off SnakeSkin Dialysis Tubing (Thermo Fisher Scientific, Waltham, MA, United States), the material was purified with a CaptoCore 400 column (Cytiva, Marlborough, MA, United States), which was equilibrated with re-dissolution buffer. The VLP capsid containing fractions in the flow-through were collected and freshly used or stored in aliquots at -30°C. Prior to the experiments, the material was thawed and filtered again through a 0.2 µm syringe filter.

### Characterization of VLP Constructs

The expression of each HBcAg VLP construct was confirmed by Western blot analysis. Re-dissolved VLPs were analyzed by SDS-PAGE and transferred onto a nitrocellulose membrane. An anti-HBcAg antibody (Abcam, Cambridge, United Kingdom) was used as the primary antibody at a 1:1,000 dilution, followed by an anti-mouse antibody (Merck Millipore, Darmstadt, Germany) at a 1:5,000 dilution. For SDS-PAGE, NuPage 4–12% BisTris protein gels, LDS sample buffer, and MES running buffer were used and run on a PowerEase 500 Power Supply (all Invitrogen, Waltham, MA, United States) at reduced mode with 50 mM DTT in the sample solution. Protein staining was performed with a Coomassie blue solution.

SEC coupled with a diode array detector was used to evaluate the phase behavior of the VLPs by quantifying and specifying differently sized species (dimers, capsids, and aggregates) and determine the loading of the VLPs with nucleic acids. An Agilent Bio SEC-5, 5 μm, 1,000 Å, 4.6 × 300 mm column (Agilent, Santa Clara, CA, United States) was used on a Vanquish UHPLC system, controlled by Chromeleon version 7.2 (both Thermo Fisher Scientific, Waltham, MA, United States). To assess the loading of VLPs with nucleic acids, the ratio of HBcAg capsid peaks at 260 and 280 nm was calculated. Scatter correction was performed as previously described for HBcAg VLPs (Porterfield and Zlotnick, 2010).

Dynamic light scattering was used to determine the hydrodynamic radius and size distribution profiles of the particles. Measurements were performed on the DynaPro Plate Reader III (Wyatt Technology, Santa Barbara, CA, United States) using a sample volume of 30 μL in a Corning 384 well plate (Corning, NY, United States). Unfiltered samples were measured six times, each measurement consisting of 25 runs of 5 s each at 25°C. The hydrodynamic radius was obtained as calculated by the Dynamics software (Version 7.10.1.21, Wyatt Technology, Santa Barbara, CA, United States). Electrophoretic mobility was measured with disposable folded capillary cells on a Zetasizer Nano ZS instrument (both Malvern Instruments Ltd., Malvern, United Kingdom). Each measurement comprised a 120 s equilibration and five runs with 15 sub-runs. The measurements were performed at 40 mV and 25°C. Zeta potential was calculated by Zetasizer software (Version 7.12, Malvern Instruments Ltd., Malvern, United Kingdom) with the measured electrophoretic mobility, a material refractive index of 1.45, absorption of 0.001, a viscosity of 0.8872 mPas, a dielectric constant of 78.54, and a Smoluchowski approximation of 1.5 (Smoluchowski, 1921).

### Purification Process Characterization

For precipitation experiments, the filtered lysate was diluted to concentrations of 0.75–1.15 M (NH_4_)_2_SO_4_ in increments of 0.05 M and 0.25% (v/v) polysorbate 20, respectively. The solution was incubated for 3 h at 4°C, spun down at 17,000 rcf for 30 min in a centrifuge 5810 R (Eppendorf, Hamburg, Germany), and the supernatant was analyzed by SDS-PAGE. According to the standard purification procedure, the pellet was resuspended with re-dissolution buffer and stirred at 150 rpm overnight at 4°C. After centrifugation and filtration, the supernatant was analyzed by SDS-PAGE. The gels were analyzed by VLP band intensity measurements using the open-source software ImageJ.

For the disassembly screening, purified VLP constructs in re-dissolution buffer, treated as biological triplicates, were diluted to 3 M, 3.5 M, or 4 M urea with a 50 mM Tris and 10 mM urea stock solution and titrated to pH 7, 7.5, or 8.0. The material was stirred at 150 rpm for 18 h at 4°C, and samples were analyzed in triplicates by SEC as described earlier. The peak areas of aggregates, capsids, and dimers were analyzed. Control runs at the high-performance liquid chromatography system without a prefilter and column were performed to determine the number of bigger aggregates, and total peak areas at 280 nm were corrected. Dimer yield was calculated by the ratio of HBcAg dimer peak area to total peak area at 280 nm (details on peak identification can be found in Supplementary Material S2).

## Results

### Characterization of HBcAg VLP Constructs

**FIGURE 2:**
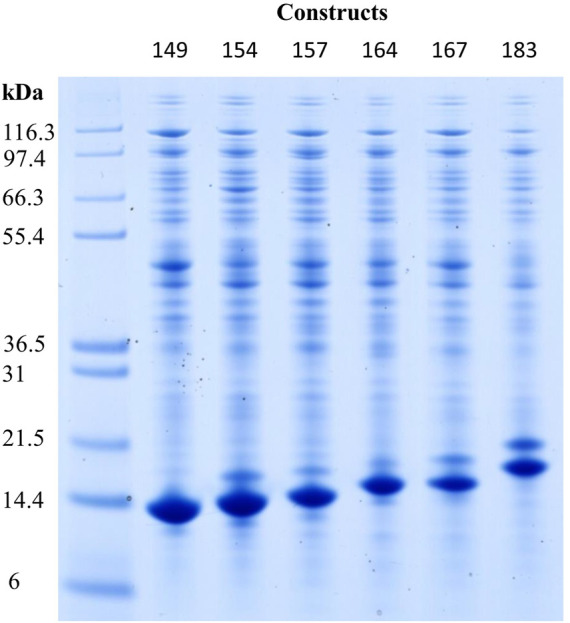
Reducing SDS-PAGE scan of HBcAg VLP construct monomers after re-dissolution in lanes two to seven, with Invitrogen Mark 12 Unstained Standard in lane 1. Molecular weights of the proteins in the standard are displayed on the left. Protein staining by Coomassie blue; HBcAg, hepatitis B core antigen; VLP, virus-like particle.

The expression of the constructs Cp149, Cp154, Cp157, Cp164, Cp167, and Cp183 was verified by Western blot analysis (for visualization see Supplementary Material S3), and different molecular weights of their monomers were displayed with SDS-PAGE (Figure 2). Cp149 showed a lane close to the 14.4 kDa marker, and Cp183 was closer to the 21.5 kDa marker. The intermediate constructs were displayed in between them in the order of their ascending lengths of amino acid sequences, respectively.

#### VLP Size and Zeta Potential Analysis

**FIGURE 3:**
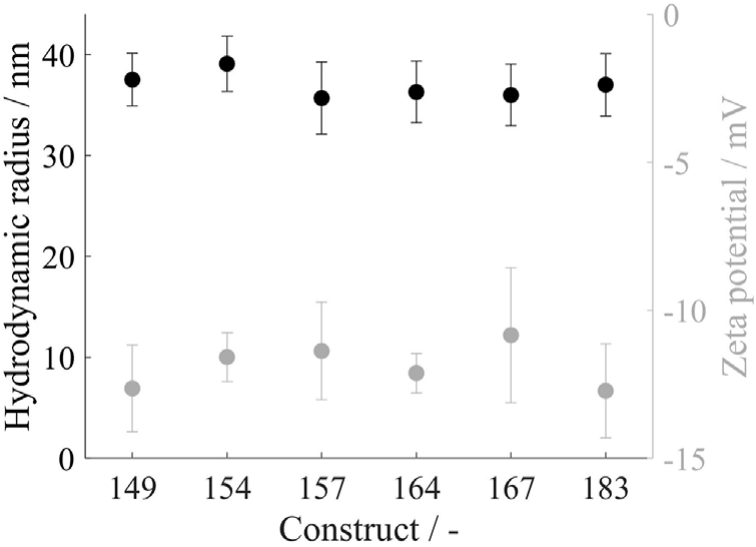
Hydrodynamic radius in black and zeta potential in grey of all VLP construct capsids. Measurements were conducted directly after CaptoCore 400 chromatography purification step. DLS measurements were conducted in six replicates and zeta potential measurements in five replicates; VLP, virus-like particle.

To determine the hydrodynamic radius of the VLP capsids, the VLP constructs were purified by the CaptoCore 400 step and directly measured by dynamic light scattering. The analysis resulted in average hydrodynamic diameters of 36.9 ± 3.2 nm, not showing distinct differences between the constructs (Figure 3). With electrophoretic mobility measurements of the same material, the zeta potential of the different constructs was investigated. For all constructs, similar zeta potentials of −11.4 ± 1.5 mV were determined (Figure 3).

#### Loading with Host Cell-Derived Nucleic Acids

**FIGURE 4:**
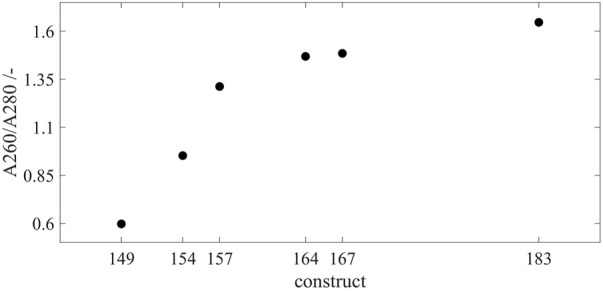
Loading of the VLP capsids with host cell-derived nucleic acids for all constructs. SEC chromatography was used to evaluate the loading of the purified VLP capsids by A260/A280 coefficient after scatter correction (Porterfield and Zlotnick, 2010). For every construct, samples were analyzed by SEC in triplicates, respectively. Error bars are negligible and therefore omitted. Details on capsid peak identification can be found in Supplementary Material S2; VLP, virus-like particle; SEC, size-exclusion chromatography.

The SEC chromatography was used to evaluate the loading of purified HBcAg VLP constructs with host cell-derived nucleic acids. The A260/A280 coefficients of the capsid peak areas were determined, respectively (Figure 4). This provides an insight into the ratio of the number of nucleic acids and proteins without performing an additional separation step of the two species to use protein-specific and nucleic acid-specific analytics separately. Cp149 without a nucleic acid binding region showed an A260/A280 of 0.60, which is considered to be pure protein (Goldfarb et al., 1951), and only a minimum or no nucleic acids are encapsulated. In general, with an increase in the length of the nucleic acid binding region, the A260/A280 coefficient rises, displaying an increased number of nucleic acids bound inside the capsids, respectively. However, this correlation shows a two-zone behavior. For the constructs Cp149, Cp154, and Cp157, with no or relatively short nucleic acid binding region, A260/A280 evinces a steep linear increase with an A260/A280 of 0.95 for Cp154 and 1.31 for Cp157. However, for the constructs with longer binding regions up to full-length wild-type, A260/A280 increases from 1.47 for Cp164 to 1.48 for Cp167 and to 1.65 for Cp183. For these constructs, the increased length of the binding domain shows less effect on the A260/A280 and thus the amount of encapsulated nucleic acids.

### Effects of VLP Constructs on the Purification Process

#### Capture by Precipitation and Re-Dissolution

**FIGURE 5:**
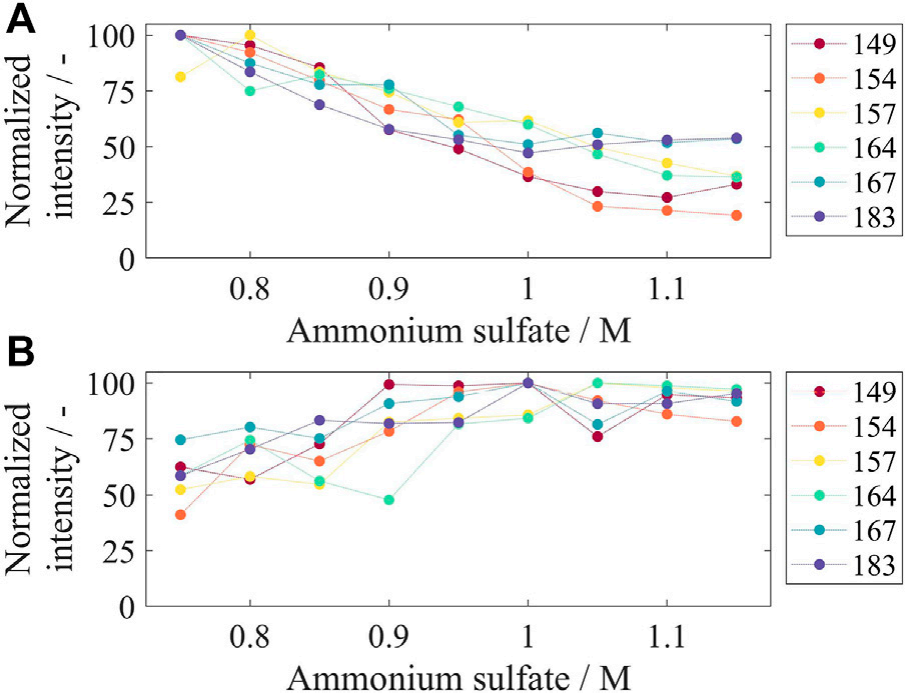
Remaining VLP constructs in precipitation supernatant **(A)** and re-dissolved VLP constructs after precipitation and re-dissolution overnight for different tested ammonium sulfate concentrations. After precipitation with different ammonium sulfate concentrations, the supernatant **(A)** and re-dissolution solution **(B)** were analyzed by SDS-PAGE, and gel scans were evaluated by the intensity of the VLP band on the gel lane with respect to the highest determined intensity for the respective construct. Corresponding gel images can be found in Supplementary Material S4; VLP, virus-like particle.

To investigate the precipitation behavior of the VLP constructs, ammonium sulfate concentrations from 0.75 M up to 1.15 M were examined. After the precipitation reaction, the supernatant was analyzed for the remaining VLPs as well as the respective re-dissolution material for precipitated and re-dissolved VLPs by SDS-PAGE. The corresponding gel images can be found in Supplementary Material S4. Figure 5A displays the intensities derived from image analysis of precipitation supernatant samples normalized to the highest determined intensity for the respective construct. For all constructs, an increase in ammonium sulfate concentration in the precipitation step reduced the amount of remaining VLPs, shown by lower intensities for the VLP fraction on the gel images of the supernatant. For Cp149 and Cp154, the intensities decrease with increasing ammonium sulfate concentrations up to 1.05 M and remain fairly constant with even higher concentrations. Cp167 and Cp183 show the same behavior with no further steady decrease in intensity equal to and higher than an ammonium sulfate concentration of 0.95 M. Considering the gel images (Supplementary Material S4) for the high ammonium sulfate concentrations with fairly stable intensities, the VLPs have been precipitated in total, and the remaining intensity signal derives from other proteins in the samples. For Cp157 and Cp164, the normalized intensities decrease for all tested ammonium sulfate concentrations which is not in alignment with the gel images where VLPs are not clearly visible for concentrations equal to and higher than 1.05 M. Further decreased intensities might be caused by other proteins co-precipitating with higher tested ammonium sulfate concentrations.

For the re-dissolved solutions, displayed in Figure 5B, high normalized intensities for all constructs were obtained for concentrations equal to and higher than 0.95 M ammonium sulfate. The overall highest intensities, considering all constructs, were at 1 M ammonium sulfate. The VLP purity after precipitation and re-dissolution ranged between 10 and 15% for all tested conditions (data not shown). For further processing, an ammonium sulfate concentration of 1 M was chosen.

In preliminary experiments, suitable buffer conditions for VLP re-dissolution were determined by investigating Tris buffer with different sodium chloride molarities. All tested conditions demonstrated the capability to re-dissolve VLPs, but 150 mM NaCl showed the highest amount of VLP capsids as opposed to disassembled or aggregated VLP capsid proteins by SEC analysis (for details on the analysis, see Supplementary Material S2), which is the desirable VLP conformation for the following purification steps.

#### Disassembly Screening

After precipitation, re-dissolution, and the separation of small contaminants by dialysis and chromatography with CaptoCore 400, the disassembly step is crucial in the purification procedure to separate encapsulated host cell-derived nucleic acids and other impurities. To investigate the phase behavior of the different constructs and the influence of encapsulated host cell-derived nucleic acids on capsid stability, purified VLP constructs were disassembled in reducing conditions, varying both the pH and the urea concentration of the liquid phase, respectively.

**FIGURE 6:**
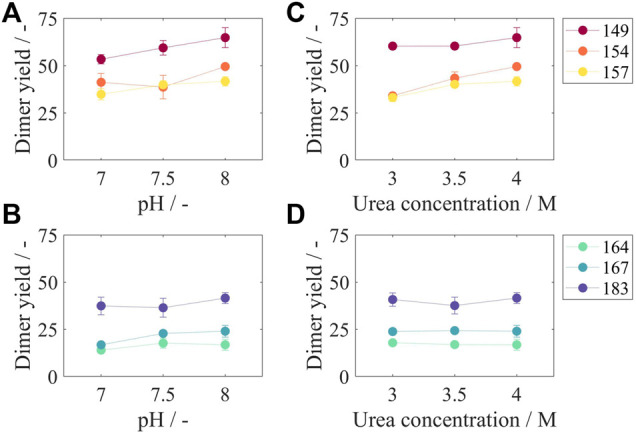
Dimer yields after disassembly reaction for different liquid phase conditions and for all constructs. In subfigures **(A)** and **(B)**, results for a urea concentration of 4 M and varying pH values are displayed. In subfigures **(C)** and **(D)**, results for a pH of eight and varying urea concentrations can be found. According to the two-zone behavior, subfigures **(A)** and **(C)** show results for Cp149, Cp154, and Cp157 and subfigures **(B)** and **(D)** for Cp164, Cp167, and Cp183, respectively. Results for 3 M urea, 3.5 M urea, pH 7, and pH 7.5 can be found in Supplementary Material S5. SEC was used to evaluate the dimer yield after the disassembly reaction. Details on capsid peak identification can be found in Supplementary Material S2; SEC, size-exclusion chromatography.

Dimer yields for all constructs and varying pH values at 4 M urea, the highest urea concentration investigated in this study, are shown in Figures 6A, B. For Cp149, an increased pH of the disassembly solution resulted in an increased dimer yield from 53.4% at pH 7 to 64.9% at pH 8. All other constructs show the same correlation, however, less pronounced, with an increase in dimer yield between 2.8% for Cp164 and 8.3% for Cp154 from pH 7 to pH 8. Additionally, dimer yields for all constructs and varying molar urea concentrations at pH 8, the highest tested pH value, are shown in Figures 6C, D. For the constructs with no or short nucleic acid binding regions Cp149, Cp154, and Cp157 (Figure 6C), increasing dimer yields from 4.9% for Cp149 up to 15.5% for Cp154 were determined when increasing the urea concentration from 3 to 4 M. For the constructs Cp164, Cp167, and Cp183 (Figure 6D), the dimer yields remained fairly constant within ±1%, comparing urea concentrations of 3 and 4 M.

Results for pH 7 and pH 7.5 and for urea concentrations of 3 and 3.5 M show equal behavior and can be found in Supplementary Material S5. Considering all tested conditions and averaging over all constructs and all pH values, the average increase in dimer yield was 4.9% when increasing the molar concentration of urea from 3 to 4 M. Likewise, averaging over all urea concentrations, the average increase of dimer yield was 7.6% when increasing the pH value from 7 to 8. However, the combination of increasing the pH and increasing the urea concentration led to an even higher increase in dimer yield of 13.3%.

When comparing the disassembly behavior of the different HBcAg VLP constructs, a two-zone behavior can again be distinguished. For all tested conditions, Cp149 without nucleic acid binding region showed the highest dimer yields with values between 48.8% (50 mM Tris, 3 M urea, pH 7) and 64.9% (50 mM Tris, 4 M urea, pH 8). As shown for varying pH values in Figure 6A and varying urea concentrations in Figure 6C, for the constructs Cp149, Cp154, and Cp157, an increased length of the nucleic acid binding region lead to lower dimer yields for almost every condition. For the constructs with longer binding regions, Cp164, Cp167, and Cp183, this correlation is reversed. As shown for varying pH values in Figure 6B and varying urea concentrations in Figure 6D, Cp164 with an intermediate length of nucleic acid binding region results in overall lowest dimer yields. However, further increased lengths of the nucleic acid binding regions up to the full-length wild-type Cp183 lead to an increase in dimer yields. This results in similar dimer yields for Cp154, Cp157, and Cp183. These findings for the results shown in Figure 6 also apply to the results for pH 7 and pH 7.5 and to urea concentrations of 3 and 3.5 M (Supplementary Material S5).

## Discussion

### Intracellular Formation and Characterization of HBcAg VLP Constructs

In this study, HBcAg VLP constructs with various lengths of the naturally occurring nucleic acid binding region were produced. To demonstrate the successful intracellular formation of all protein variants, Western blot analysis and SDS-PAGE were performed. The resulting monomer sizes of the constructs under denaturing conditions are consistent with the size values given in the literature for Cp183 of 21 kDa (Wynne et al., 1999; Bin Mohamed Suffian et al., 2017) as well as the shorter migration of the truncated constructs (Tan et al., 2003). Despite the different monomer sizes, assembled capsids showed a hydrodynamic radius of 36.9 ± 3.2 nm for all constructs, which is in alignment with the reported 36 nm for Cp183 (Porterfield et al., 2010). In the literature, *T* = 4 and *T* = 3 icosahedral structures of HBcAg VLP capsids with differences in their sizes were reported (Wingfield et al., 1995; Conway et al., 1997; Conway et al., 1998). However, the DLS measurement was unable to resolve these different HBcAg species within this study and in the literature (Petrovskis et al., 2021). The independence of the capsid size from HBcAg VLP constructs has already been suggested in the literature by evaluating transmission electron microscope pictures and measuring electrophoretic mobilities and thereby determining a hydrodynamic radius of about 38 nm for all investigated HBcAg protein genotypes (Petrovskis et al., 2021). This underlines the structural homogeneity of HBcAg VLPs with icosahedral structures (Venkatakrishnan and Zlotnick, 2016), which is a major advantage of VLPs over other nucleic acid carrier structures investigated in the literature (Rohovie et al., 2017). Furthermore, the zeta potential was also fairly constant for all constructs despite the different lengths of the nucleic acid binding region. The positive charges of the arginine-rich binding region do not seem to affect the negative surface charge, likely because of its orientation toward the inside of the capsid (Venkatakrishnan and Zlotnick, 2016) and due to charge compensation by the encapsulated nucleic acids (Le Pogam et al., 2005).

A260/A280 ratios for the capsid peaks in the SEC chromatograms were determined for all constructs to assess the amount of encapsulated nucleic acids. For Cp149, the A260/A280 was 0.60, which is comparable to previously reported values of 0.61 (Hillebrandt et al., 2021) and 0.67 (Zhang et al., 2021) before further purification. For pure protein, A260/A280 ratios of 0.6 are assumed (Goldfarb et al., 1951), and accordingly, for purified HBcAg capsid protein, an A260/A280 ratio of about 0.6 was reported after performing the proposed scatter correction (Porterfield and Zlotnick, 2010). Diverging from this, even lower A260/A280 ratios of 0.55 (Hillebrandt et al., 2021) and 0.57 (Zhang et al., 2021) were reported after disassembly followed by cross-flow filtration or affinity chromatography, demonstrating that there are still a few nucleic acids encapsulated in Cp149 capsids, which are then depleted by the mentioned subsequent purification steps. Resulting from this, the scatter-corrected A260/A280 of 0.60 demonstrates that nucleic acids are encapsulated, even without having a nucleic acid binding region within the Cp149 capsid. However, assessed on the basis of the absorbance ratios, the amount of encapsulated nucleic acids in Cp149 is low compared to the Cp183 with the full-length wild-type nucleic acid binding region (A260/A280 of 1.65), which is in line with previously described results in the literature based on cryogenic electron microscopy analysis of the two constructs (Crowther et al., 1994; Zlotnick et al., 1997). Considering all investigated constructs with varying lengths of the nucleic acid binding region, the A260/A280 ratios show that the longer the nucleic acid binding region, the more nucleic acids are encapsulated, which was also observed previously by cryogenic electron microscopy (Liu et al., 2010). Interestingly, the analysis of the loading of the nucleic acids did not result in a linear correlation between the encapsulation of host cell-derived nucleic acids after intracellular formation and the length of or the number of positive charges within the nucleic acid binding region. A two-zone behavior was hereby distinguishable. In one zone, including the constructs with no or short binding regions, an increased length of the binding region has a significant impact on the amount of encapsulated nucleic acids. Whereas for the constructs with longer binding regions in the second zone, a longer binding region has little effect on the amount of encapsulated nucleic acids.

This two-zone behavior affects the charge ratios between the positive charges within the nucleic acid binding region and the negative charges derived from the nucleic acids within the capsids, which were identified to influence the capsid stability (Le Pogam et al., 2005; Newman et al., 2009). It was hypothesized that a balanced charge density inside the capsid leads to stable capsid structures (Le Pogam et al., 2005). Additionally, nucleic acids bound to the binding region of the HBcAg VLP constructs form an inner shell within the capsid (Zlotnick et al., 1997; Liu et al., 2010) and thus are assumed to stabilize its structure.

Moreover, when considering the loading with host cell-derived nucleic acids as an indicator for the capability of the HBcAg VLP constructs to encapsulate therapeutic nucleic acids later in the process, the nucleic acid binding region seems to be important for an efficient encapsulation. However, the two-zone behavior with the non-linear correlation between the length of the nucleic acid binding region and the amount of encapsulated nucleic acids reveals that an intermediate length of the nucleic acid binding region might be sufficient for effective encapsulation of the therapeutic nucleic acid. This is particularly interesting due to stability problems after host cell-derived nucleic acid depletion for Cp183 in the later purification process (Porterfield et al., 2010), as well as for Cp164 and Cp167 (Le Pogam et al., 2005) were reported, whereas Cp154 showed no such stability problems (Le Pogam et al., 2005).

### Capture by Precipitation and Re-Dissolution

Effects of the different HBcAg VLP constructs and especially their characteristics on production and purification steps such as selective precipitation, re-dissolution, and disassembly were investigated in this study.

For all constructs, an ammonium sulfate concentration of 1.05 M was sufficient to fully precipitate the VLPs from the clarified lysate. For Cp167 and Cp183, even a concentration of 0.95 M was enough for precipitation. However, this difference in needed ammonium sulfate concentrations within the investigated constructs, here, is small compared to the big leap to the reported necessary concentration of only 0.15 M for a chimeric HBcAg without nucleic acid binding region, and thus a chimeric version of Cp149 (Hillebrandt et al., 2020). It seems like changes on the surface of the HBcAg VLPs such as the foreign epitopes have a greater influence on the susceptibility to ammonium sulfate as a precipitating agent than the different lengths of the nucleic acid binding regions and bound nucleic acids that are both placed within the VLP capsids (Zlotnick et al., 1997; Liu et al., 2010). This could also be the reason for another divergence in the findings for the chimeric HBcAg VLP and the here investigated constructs. Testing ammonium sulfate concentrations in identical increments of 50 mM, the precipitation for the chimeric VLP occurred relatively abrupt within two increments at most for every reported experiment (Hillebrandt et al., 2020), whereas for the here tested constructs, the precipitation behaved more dynamically with a broader range of at least four increments, thus, 200 mM ammonium sulfate, where only a fraction of VLPs was precipitated, and the rest remained in solution.

After investigating the precipitation behavior of the different constructs, an appropriate precipitation condition for further processing was also determined. Due to the relatively small differences in the precipitation behavior of the various constructs, the same precipitation condition with 1 M ammonium sulfate was selected for further processing, showing the highest amounts of VLPs re-dissolved in total. As explained, the re-dissolution buffer was also uniform for all constructs. A detailed investigation of the re-dissolution behavior similar to the disassembly experiments was not found to be practicable because a precise analysis of the individual components, especially the determination of the dimer content, was unfeasible due to the presence of impurities of a similar size giving overlapping chromatography peaks with the analytical setup used in this study. The following preparative chromatography step separated the interfering impurities predominantly and enabled the detailed analysis of the different VLP compositions after the disassembly reaction by analytical size-exclusion chromatography.

### Effects of Liquid Phase Conditions on Disassembly Yield

The disassembly reaction is crucial for effectively separating encapsulated impurities (Hillebrandt et al., 2021), improving structural integrity (Zhao et al., 2012), and separating bound nucleic acids (Porterfield et al., 2010; Strods et al., 2015; Zhang et al., 2021). Therefore, a comprehensive disassembly screening was conducted for the different constructs in this study. The disassembly experiments showed the expected correlation between liquid phase conditions and dimer yields. In the tested range, higher dimer yields were observed for both higher pH values and higher urea concentrations. Synergistic effects of pH and urea concentration were observed, as already described for Cp149 in an earlier publication of our group (Hillebrandt et al., 2021). High pH, low ionic strength, and the addition of reducing agents such as urea or guanidine hydrochloride are widely used in the literature for the disassembly of HBcAg (Porterfield et al., 2010; Strods et al., 2015; Bin Mohamed Suffian et al., 2017; Hillebrandt et al., 2021; Zhang et al., 2021) and other VLPs (McCarthy et al., 1998; Mach et al., 2006). Commonly, a concentration of 4 M urea is used to disassemble HBcAg Cp149 and Cp183 (Porterfield et al., 2010; Zhang et al., 2021). For a chimeric version of Cp149, 4 M urea was reported to be the optimal urea concentration for the disassembly reaction (Hillebrandt et al., 2021). Both reported findings of 4 M urea as a suggested condition for disassembly are in alignment with the results for the different HBcAg VLP constructs presented in this study. Urea concentrations above 4 M can lead to unfavorable protein denaturation and aggregation (Zhang et al., 2021). However, the conditions investigated in this study with urea concentrations from 3 to 4 M and varying pH values all lead to a merely partial disassembly into dimers and also to aggregation and residual capsids, with a maximal dimer yield of 64.9% for Cp149. This observation has been reported before for Cp149 and a chimeric version of Cp149, with similar dimer yields of 71 and 69%, respectively (Hillebrandt et al., 2021). These slightly better yields compared to the results shown in this study can be explained by differences in the tested pH range, the experimental setup with different dilution strategies, and observed reaction times, which have an influence on the dimer yields.

### Effects of HBcAg VLP Constructs and Loading on Disassembly Yield

In addition to the effects of liquid phase conditions on the disassembly reaction, great differences in the disassembly behavior of the investigated HBcAg VLP constructs were observed. Dimer yields correlate with the two-zone behavior for loading of the different constructs. For the constructs Cp149, Cp154, Cp157, and Cp164 with an increased length of the nucleic acid binding region and a rising amount of bound nucleic acids, dimer yields decline steadily. It can be concluded that for these constructs, the addition of the nucleic acid binding region and thus the elicited binding of host cell-derived nucleic acids as an inner shell within the capsid (Zlotnick et al., 1997; Liu et al., 2010) increase capsid stability, which impedes the disassembly of the VLP capsids into dimers. As the loading results show, the further elongation of the nucleic acid binding region has little effect on the loading of the capsids. However, the insertion of more positive charges within the nucleic acid binding region appears to impact the capsid stability (Le Pogam et al., 2005) and thus the disassembly reaction in a reversed manner. For the constructs Cp164, Cp167, and Cp183, with an increased length of the nucleic acid binding region, dimer yields rise steadily, as opposed to the correlation for the constructs with no or short binding regions. It can be assumed that hereby, the insertion of additional positively charged arginines is not compensated by the small amount of more bound nucleic acids and their negative charges. This has been previously described as part of a charge balance hypothesis (Le Pogam et al., 2005). These surplus positive charges lead to repulsive forces within the VLP capsids and thus destabilize the capsid and enhance disassembly (Newman et al., 2009). This effect is clearly depicted by the rising dimer yields for the constructs Cp164, Cp167, and Cp183 found in this study. The investigation of the disassembly behavior of the various HBcAg VLP constructs with different loads of host cell-derived nucleic acids demonstrated both capsid-stabilizing effects of the bound nucleic acids and capsid-destabilizing effects of the positive charges within the nucleic acid binding region. Moreover, it demonstrated the significant influence of these stabilizing and destabilizing effects on the disassembly reaction and dimer yields of the different constructs.

## Conclusion

In this study, HBcAg VLP constructs with variable lengths of the natural nucleic acid binding, the truncated wild-type Cp149, the wild-type Cp183, and four intermediates were produced and characterized. The investigated constructs showed no considerable differences in capsid sizes and zeta potential, whereas the length of the nucleic acid binding region demonstrated a vast effect on the amount of host cell-derived bound nucleic acids classified as two-zone behavior. Cp149 showed merely a minimal amount of encapsulated nucleic acids. The insertion of the nucleic acid binding region leads to a steep increase in the amount of bound nucleic acids for constructs in the zone with short nucleic acid binding regions. In the other zone, from construct Cp164, a further increased length of the nucleic acid binding region showed a lower effect on the increase in the amount of encapsulated nucleic acids. Furthermore, the effects of the various constructs and their characteristics on purification steps were investigated. The capturing by precipitation and re-dissolution was scarcely affected by the different constructs and the loading with host cell-derived nucleic acids. However, the disassembly reaction appeared to be immensely dependent on the construct and its loading. The comprehensive screening of the disassembly reaction for all constructs and varying liquid phase conditions revealed a complex interplay of capsid-stabilizing effects of the bound nucleic acids and capsid-destabilizing effects, arising from repulsive forces caused by the positive charges of the nucleic acid binding region following the two-zone behavior of the loading of the constructs. The capsid stability significantly influences the disassembly reaction and achievable dimer yields. Therefore, the highest dimer yields were observed for high pH and high urea concentrations in the investigated range.

## Data Availability

The raw data supporting the conclusion of this article will be made available by the authors, without undue reservation.

## Abbreviations

HBcAg, hepatitis B core antigen; PCR, polymerase chain reaction; SEC, size-exclusion chromatography; and VLP, virus-like particle.
