# Supplementary material for: Effects of Different Lengths of a Nucleic Acid Binding Region and Bound Nucleic Acids on the Phase Behavior and Purification Process of HBcAg Virus-Like Particles
Source: Front Bioeng Biotechnol. 2022 Jul 1;10:929243. doi: 10.3389/fbioe.2022.929243 (PMC9283707; doi:10.3389/fbioe.2022.929243)
Supplement: Supplementary file 1 [file DataSheet1.docx]

Supplementary Material

Angela Valentic, Jakob Müller, Jürgen Hubbuch

*Institute of Process Engineering in Life Sciences, Section IV: Biomolecular Separation Engineering, Karlsruhe
Institute of Technology (KIT), Fritz-Haber-Weg 2, 76131 Karlsruhe, Baden-Württemberg, Germany*

S1 Oligonucleotides for Plasmid Cloning

The plasmids coding for Cp154, Cp157, Cp164 and Cp167 were made by modification of the pET11c plasmid coding Cp149. Regions encoding for the Cp149 on the pET11-based vector were amplified using overlapping oligonucleotides to introduce site-directed mutagenesis using the polymerase chain reaction. The wild-type HBcAg Cp183 was obtained by amplifying the Cp167 plasmid in the same manner. The forward and reverse primers used for the PCR reaction for the production of the different constructs, respectively, can be found in the **Table S1.1**.

**Table S1.1.** Overlapping oligonucleotides used for site-directed mutagenesis amplification used for the production of the different constructs

| Primers | Overlapping oligonucleotides 5’- 🡪 -3’ |
| --- | --- |
| Cp154 forward | AGGCGAAGAGGTCGTTAGGATCCGGCTGCTAACAAAGCCCGAAA |
| Cp154 reverse | AGGGAAGAGGTCGTTAGAACAACCGTAGTCTCCGGAAGTGTC |
| Cp157 forward | AGGCGAAGAGGTCGTTCGCCCCGGTAGGATCCGGCTGCTAACAAAGCCCGAAA |
| Cp157 reverse | AGGCGAAGAGGTCGTTCGCCCCGGAACAACCGTAGTCTCCGGAAGTGTC |
| Cp164 forward | TCGCCCCGGAGACGCACGCCCTCGCCCCGTTAGGGATCCGGCTGCTAACAAAGCCCGAA |
| Cp164 reverse | AGGCGAAGAGGTCGTTCGCCCCGGAGACGCACGCCCTCGCCCCGTTAGAACAACCGTAGTCTCCGGAAGTGTC |
| Cp167 forward | AGGCGAAGAGGTCGTTCGCCCCGGAGACGCACGCCCTCGCCCCGTAGGCGAAGATAGGATCCGGCTGCTAACAAAGCCCGAA |
| Cp167 reverse | AGGCGAAGAGGTCGTTCGCCCCGGAGACGCACGCCCTCGCCCCGTAGGCGAAGAAACAACCGTAGTCTCCGGAAGTGTC |
| Cp183 forward | CGCCGACGTTCGCAGTCACGGGAGAGCCAATGCTAGGATCCGGCTGCTAACAAAGCCC |
| Cp183 reverse | TGACTGCGAACGTCGGCGGCGAGGACTTTGTGATCTTCGCCTACGGGGCGAGGGCGTG |

S2 SEC analysis

Size-exclusion chromatography (SEC) coupled with a diode array detector was used to evaluate the phase behaviour of the VLPs by quantifying and specifying differently sized species (dimers, capsids, aggregates) and determine the loading of the VLPs with nucleic acids. A typical chromatogram is shown in **Figure S2.1.** For samples after the disassembly reaction three main peaks were detected. Peak was defined as aggregates, Peak 2 HBcAg capsids and Peak 3 as HBcAg dimers. For the calculation of the dimer yield additionally control runs at the high-performance liquid chromatography system without a prefilter and column were performed to determine the amount of bigger aggregates that cannot appear in the SEC chromatogram due to their size. For the analysis of the loading, Peak 2 at 280 nm and 260 nm was consulted to calculate the A260/A280 coefficient.


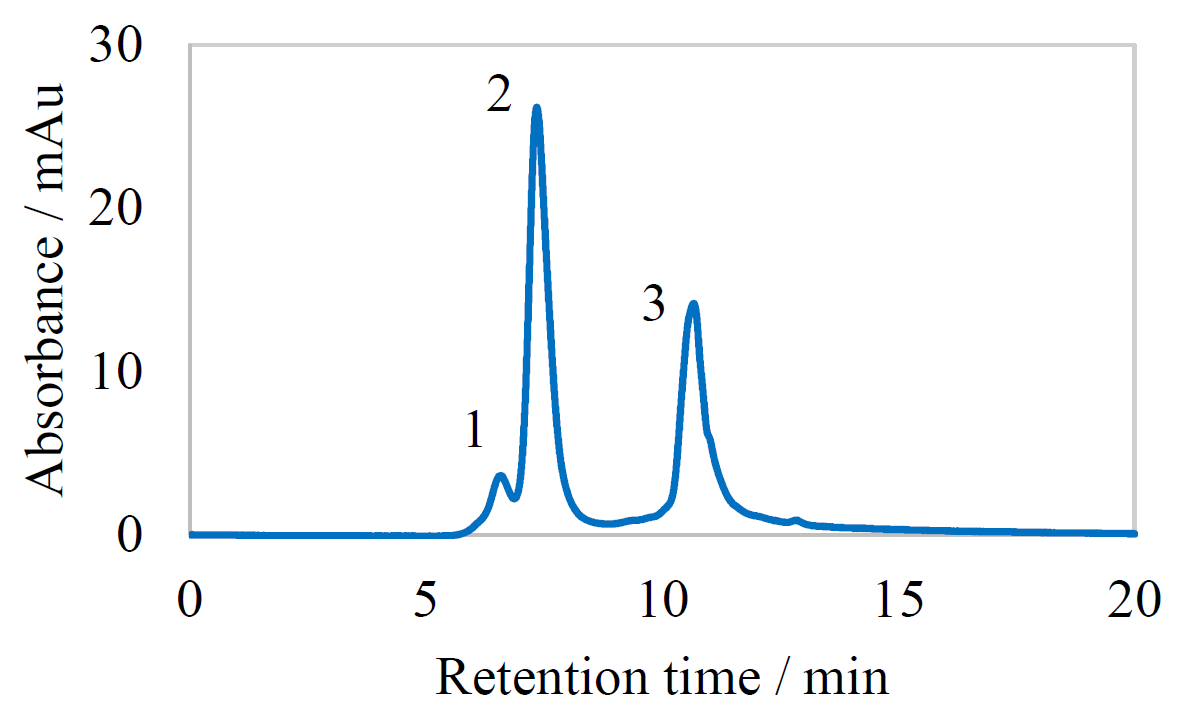


**Figure S2.1.** Size exclusion chromatography chromatogram VLP solution showing absorbance at 280 nm over retention time. Peak 1 represents VLP aggregates, peak 2 VLP capsids and peak 3 dimers; SEC, size-exclusion chromatography; VLP, virus-like particle

A detailed investigation of the re-dissolution behaviour similarly to the disassembly experiments was not found to be practicable, because a precise analysis of the individual components, especially the determination of the dimer content, were unfeasible due to impurities with the similar size giving overlapping chromatography peaks with the analytical setup used in this study.

S3 Results of Western Blot Analysis

The expression of the constructs Cp149, Cp154, Cp157, Cp164, Cp167 and Cp183 was verified by Western Blot analysis. Results are displayed in **Figure S3.1**.


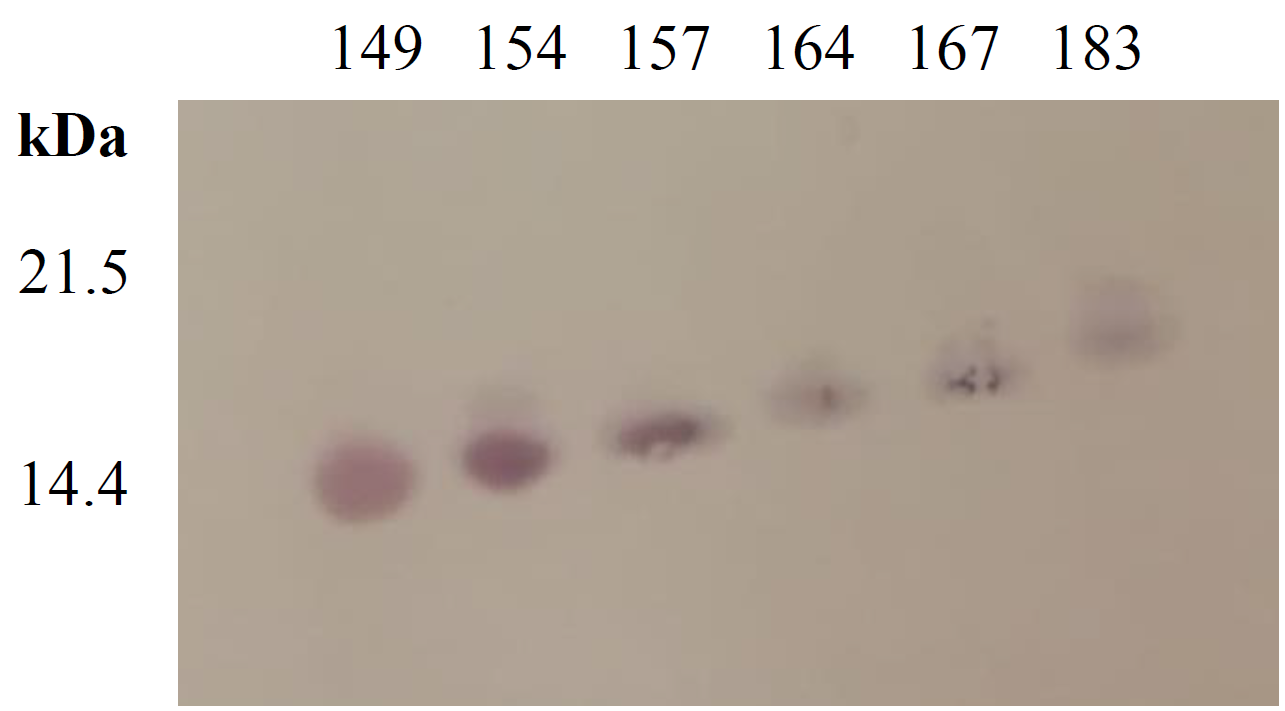


**Figure S3.1.** Scan of Western Blot analysis. Re-dissolved VLPs were analysed by SDS-PAGE and transferred onto a nitrocellulose membrane. As primary antibody an anti-HBcAg antibody was used, followed by an anti-mouse; HBcAg, Hepatitis B core Antigen

S4 SDS-PAGE Scans for Precipitation and Re-dissolution experiments

To investigate the precipitation behavior of the VLP constructs, ammonium sulfate concentrations from 0.75 M up to 1.15 M were examined. After the precipitation reaction, the supernatant was analysed for the remaining VLPs as well as the respective re-dissolution material for precipitated and re-dissolved VLPs by SDS-PAGE. The corresponding gel images can be found in the **Table S4.1**.

**Table S4.1.** SDS-PAGE scans of the supernatant after precipitation (left column) and the respective re-dissolution material (right column) for. Protein standard in lane 1, and samples for ammonium sulfate concentrations from 0.75 M up to 1.15 M in lanes 2 to 11, respectively. For re-dissolved Cp 154 lane 11 with 1.15 M is missing on the gel.

| Precipitation supernatant |  | Re-dissolution material |
| --- | --- | --- |
| 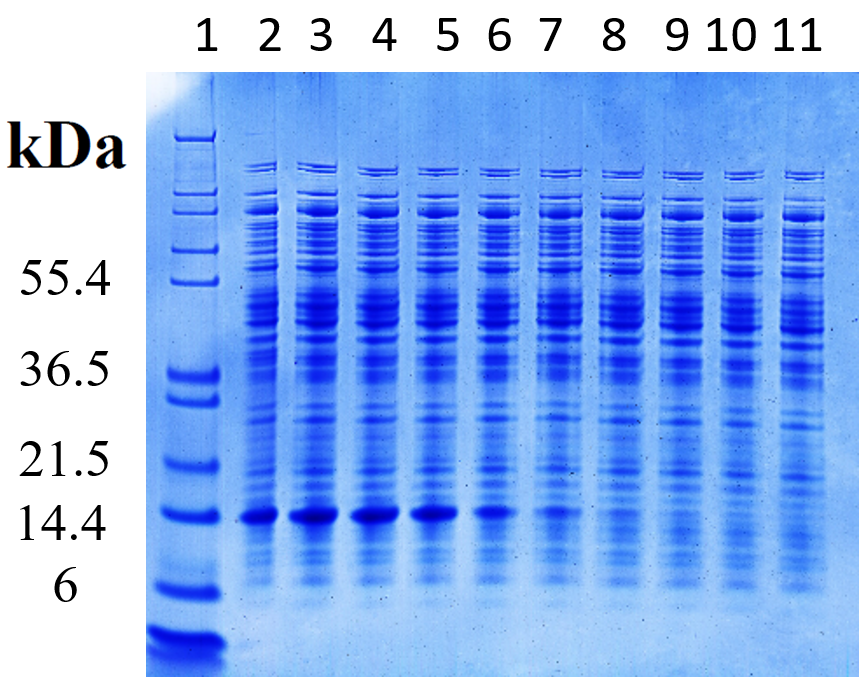 | **149** | 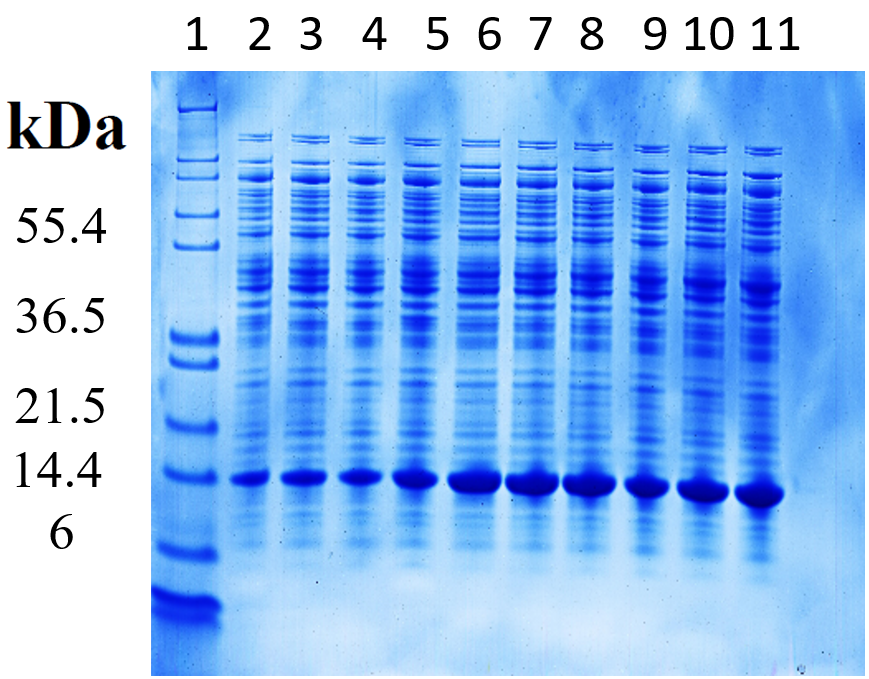 |

| Precipitation supernatant |  | Re-dissolution material |
| --- | --- | --- |
| 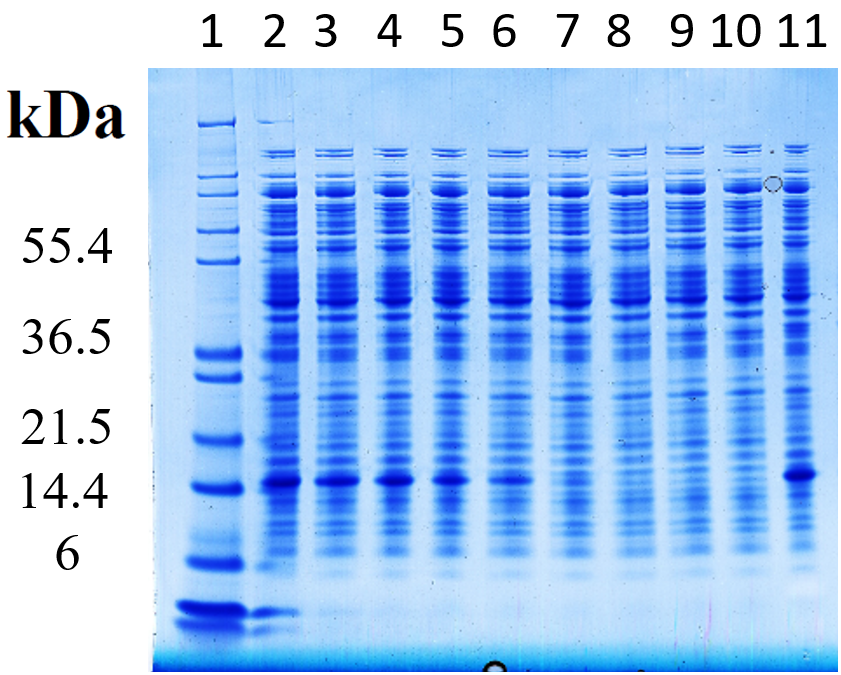 | **154** | 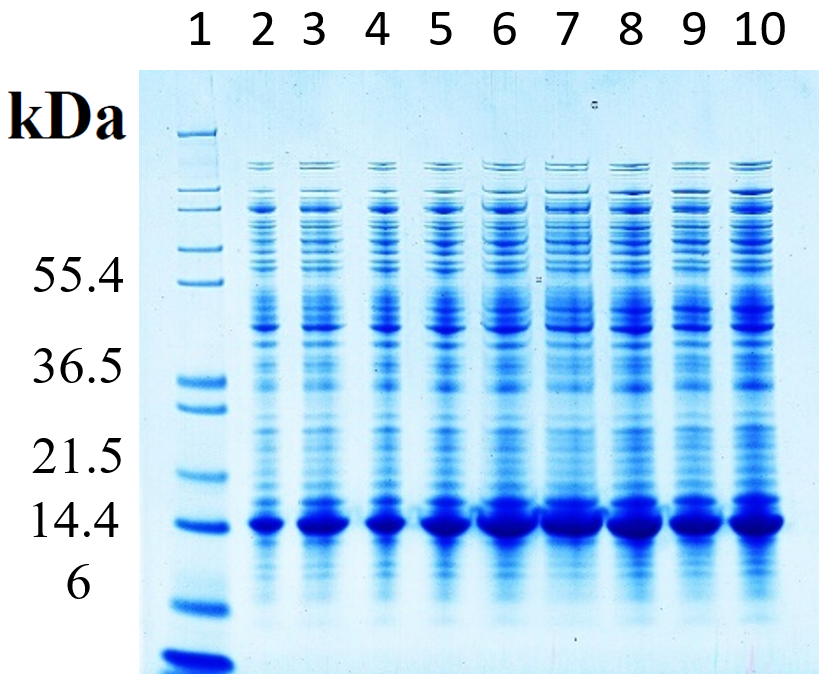 |
| 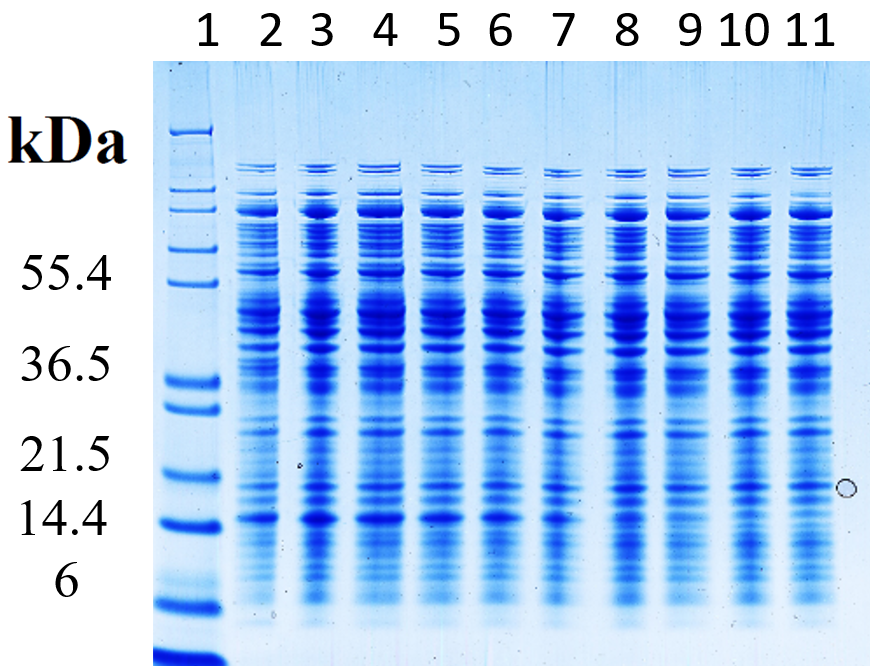 | **157** | 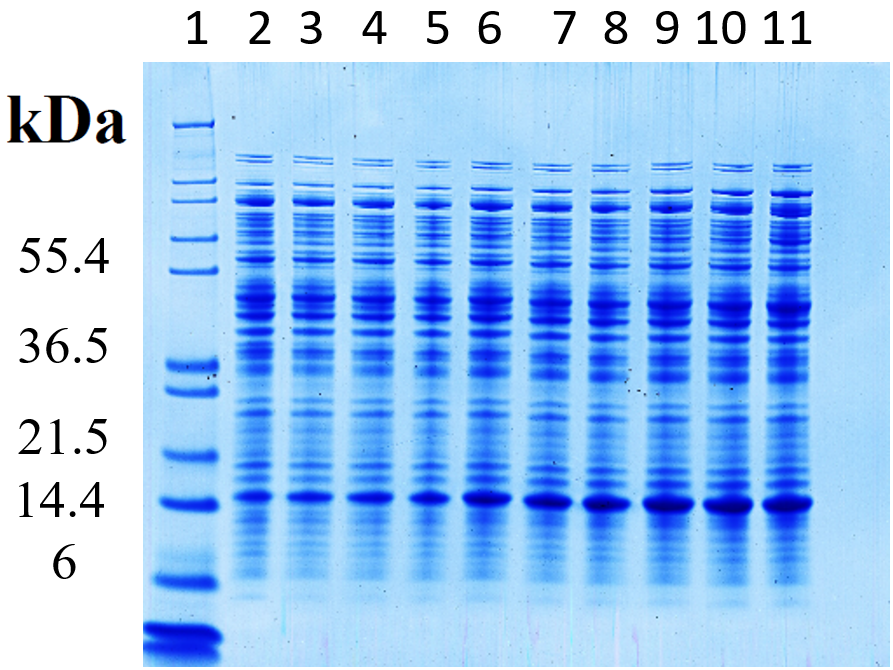 |
| 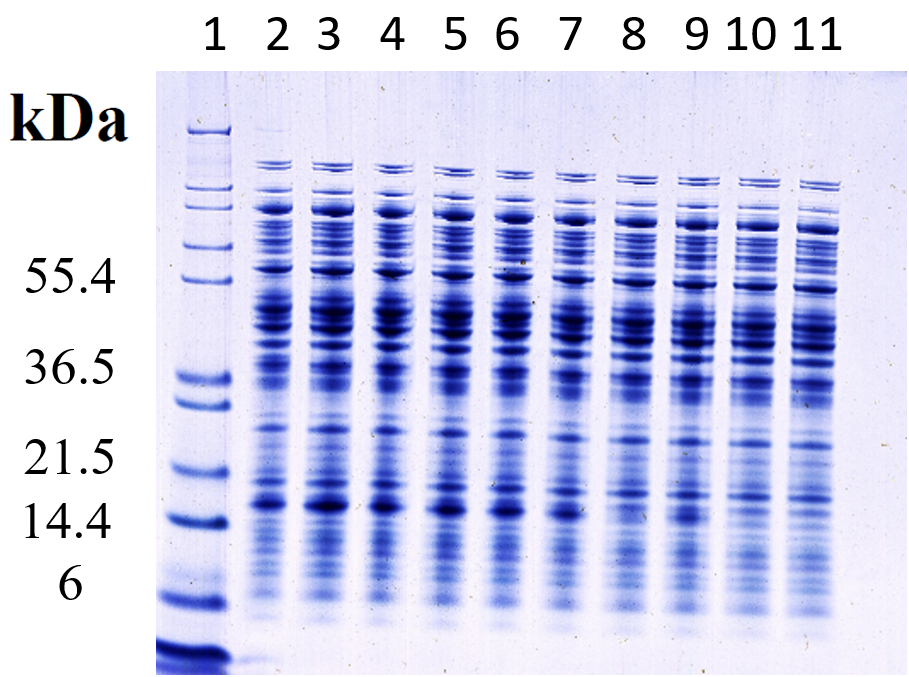 | **164** | 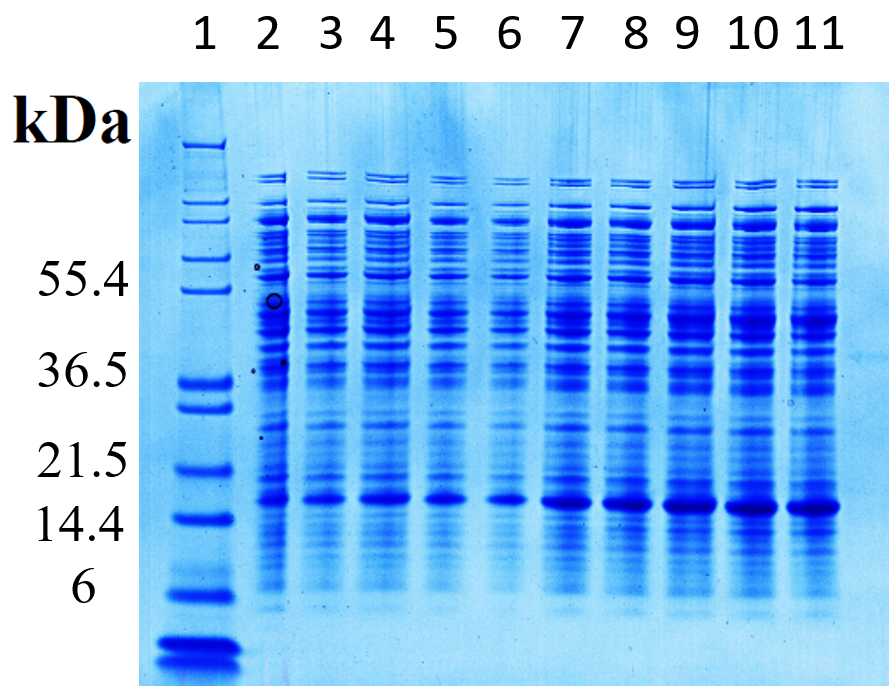 |
| 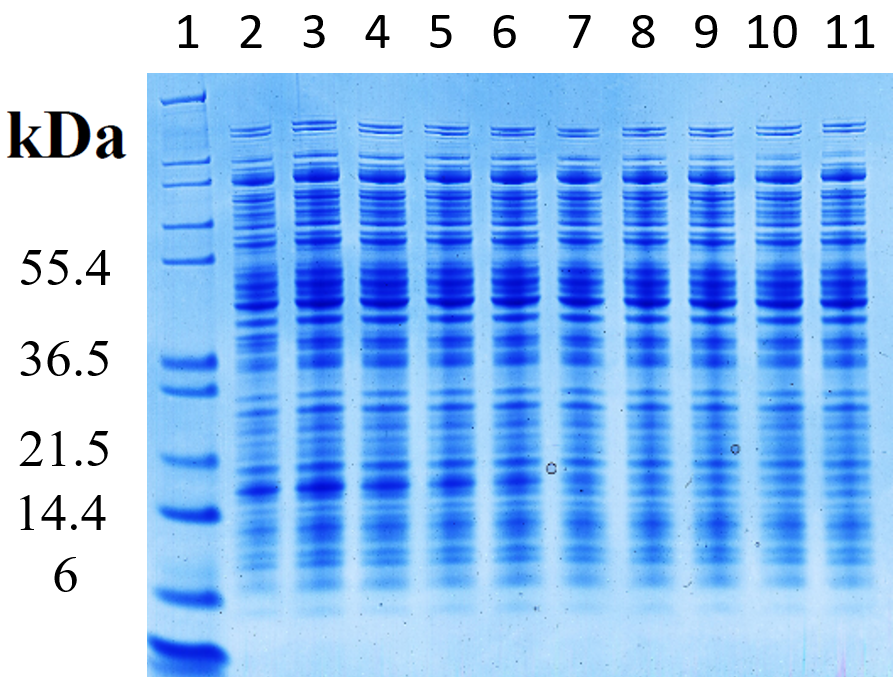 | **167** | 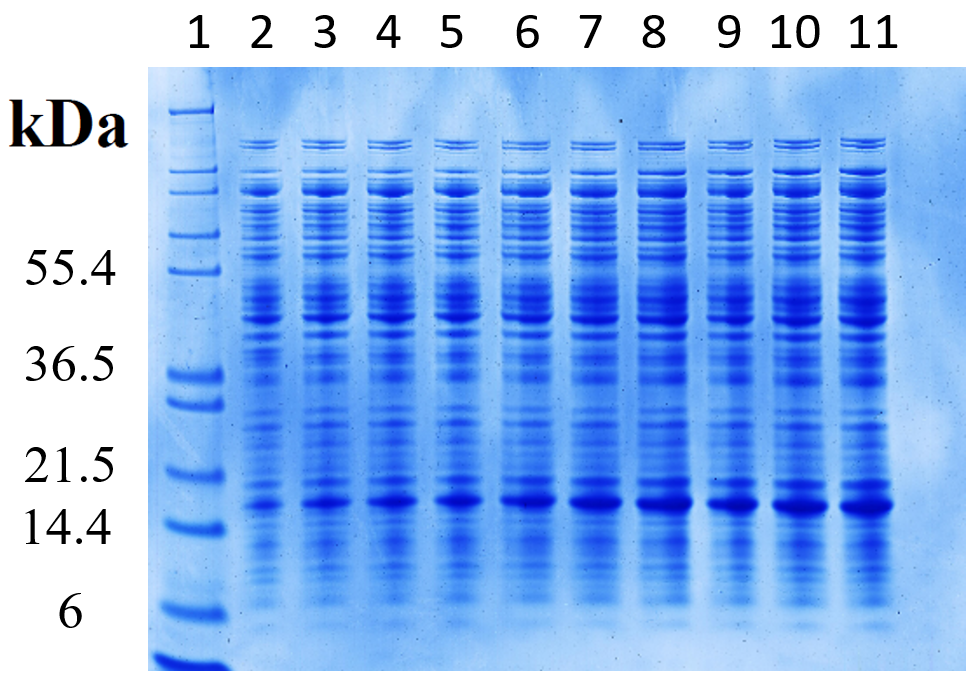 |

| Precipitation supernatant |  | Re-dissolution material |
| --- | --- | --- |
| 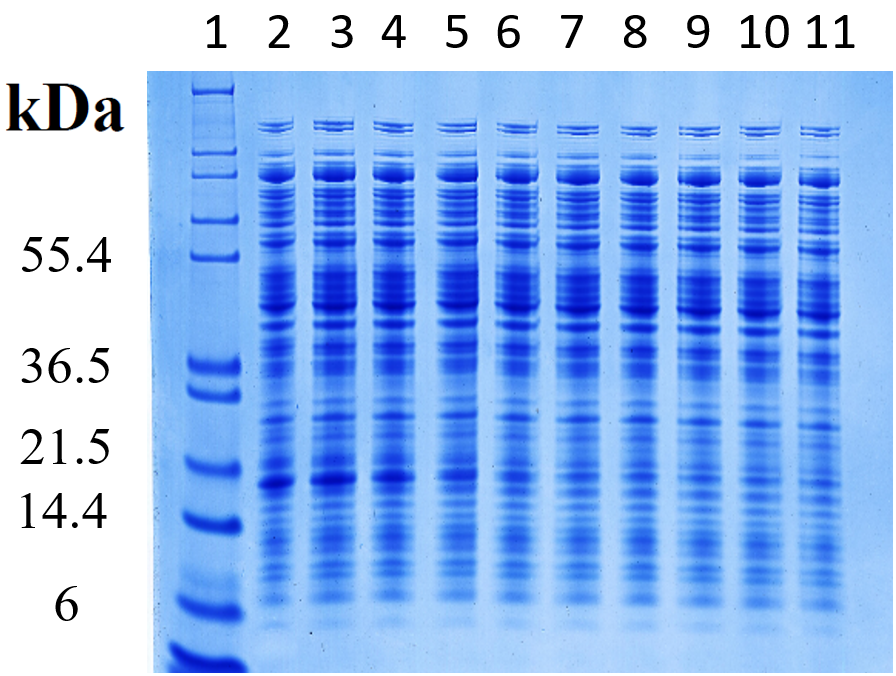 | **183** | 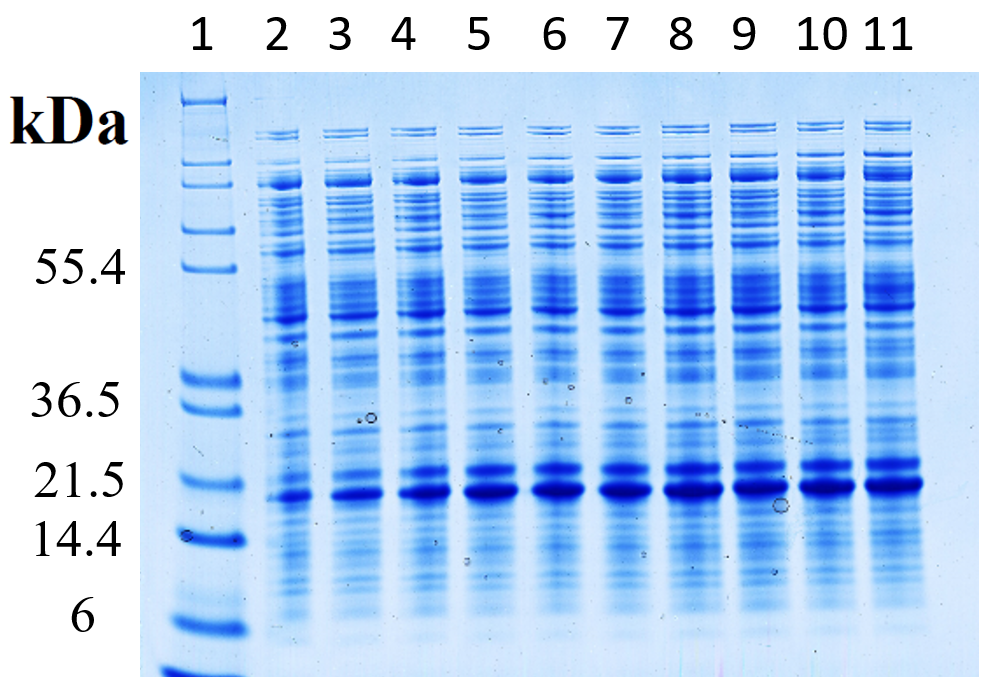 |

S5 Disassembly Screening Results for pH 7, pH 7.5, 3 M urea and 3.5 M urea

Dimer yields for the are displayed in **Table S5.1**.

**Table S5.1.** Dimer yields after disassembly reaction pH 7, pH 7.5, 3 M urea and 3.5 M urea conditions and for all constructs.

| pH 7 | |
| --- | --- |
| 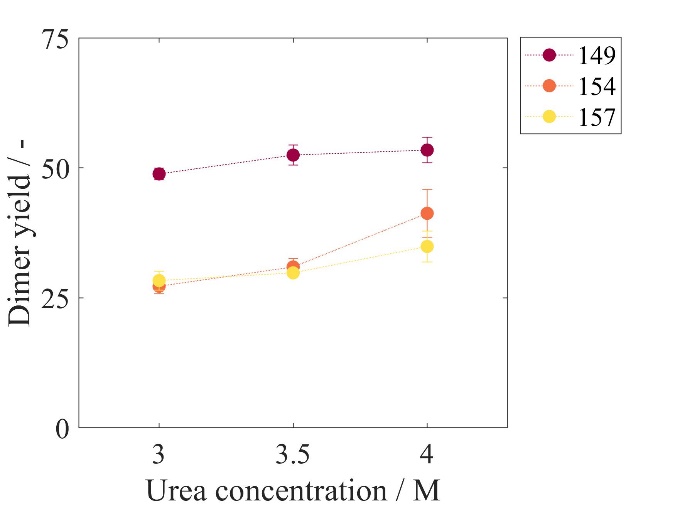 | 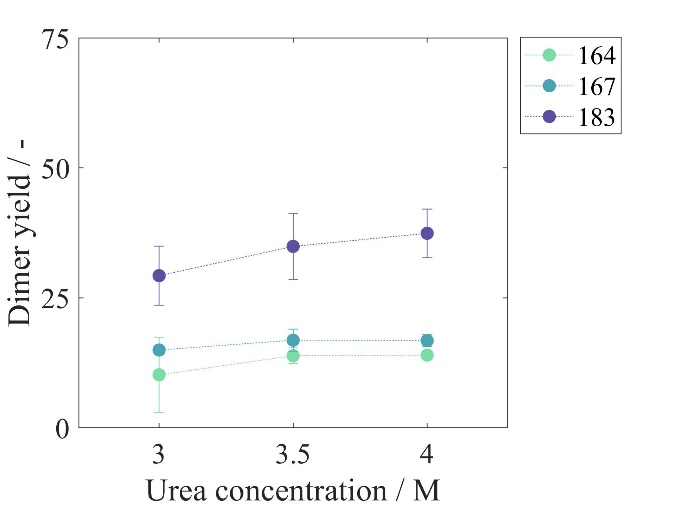 |

| pH 7.5 | |
| --- | --- |
| 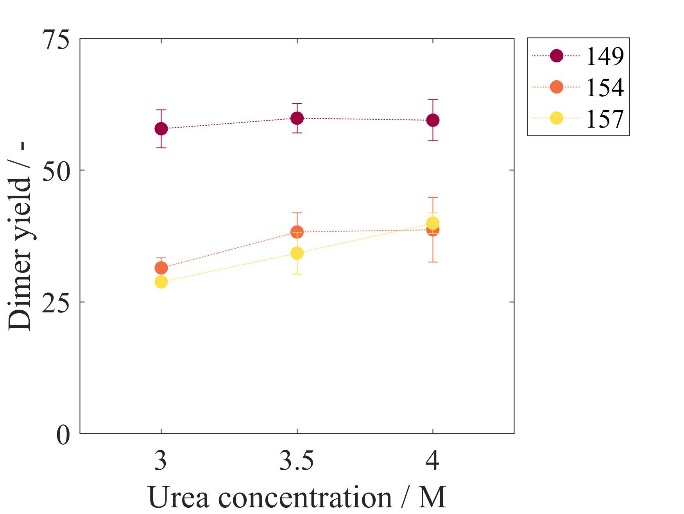 | 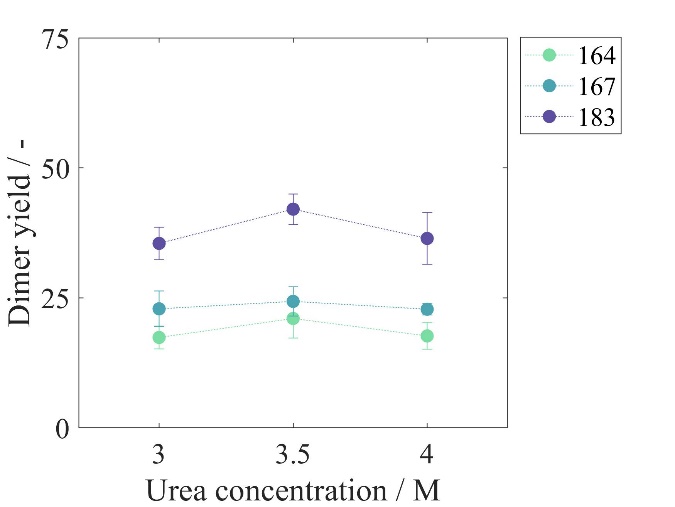 |
| 3 M urea | |
| 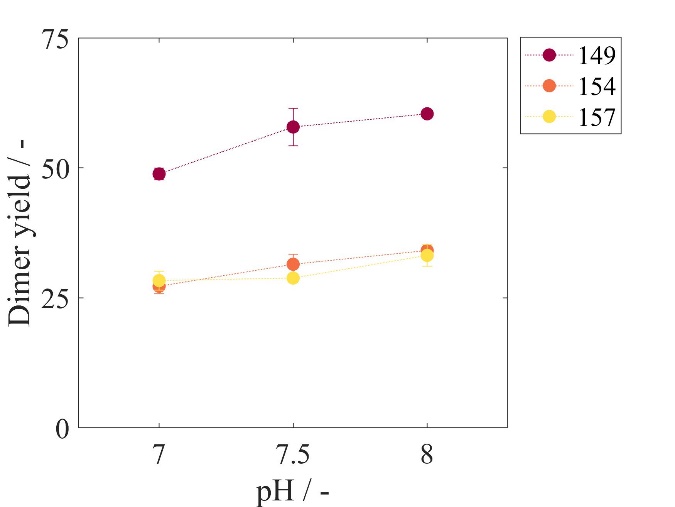 | 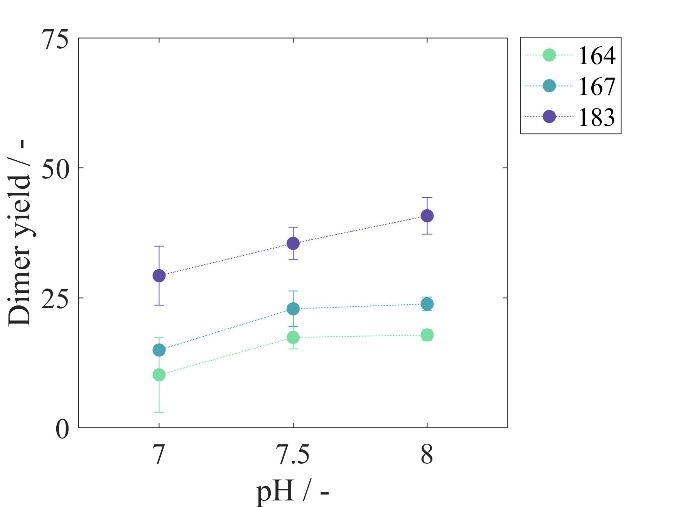 |
| 3.5 M urea | |
| 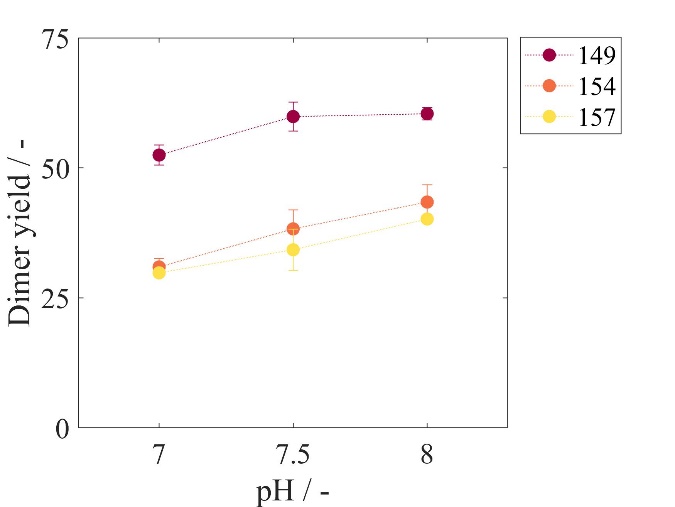 | 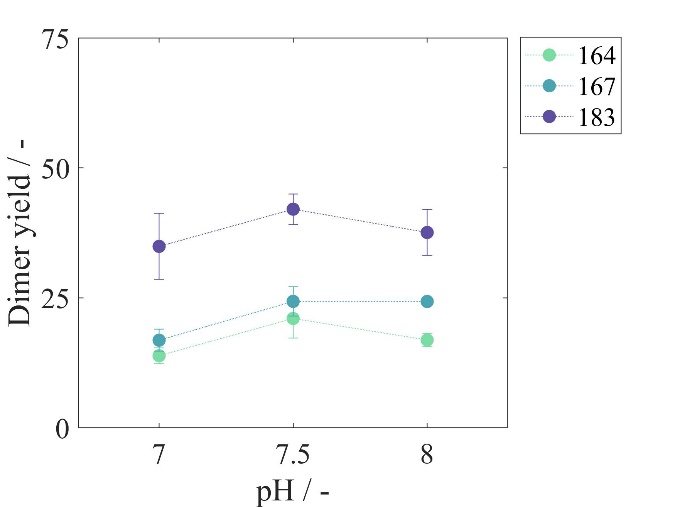 |
